# Implanting mechanically reprogrammed fibroblasts for aged tissue regeneration and wound healing

**DOI:** 10.1111/acel.14032

**Published:** 2023-11-27

**Authors:** Bibhas Roy, Tina Pekec, Luezhen Yuan, G. V. Shivashankar

**Affiliations:** ^1^ Division of Biology and Chemistry Paul Scherrer Institute Villigen Switzerland; ^2^ Department of Health Sciences and Technology ETH Zurich Zurich Switzerland

**Keywords:** ECM remodeling, human skin model, mechanical reprogramming, skin rejuvenation, wound healing

## Abstract

Cell‐based therapies are essential for tissue regeneration and wound healing during aging. Autologous transplantation of aging cells is ineffective due to their increased senescence and reduced tissue remodeling capabilities. Alternatively, implanting reprogrammed aged cells provides unique opportunities. In this paper, we demonstrate the implantation of partially reprogrammed aged human dermal fibroblasts into in vitro aged skin models for tissue regeneration and wound healing. The partially reprogrammed cells were obtained using our previously reported, highly efficient mechanical approach. Implanted cells showed enhanced expression of extracellular matrix proteins in the large area of aged tissue. In addition, the implanted cells at wound sites showed increased extracellular matrix protein synthesis and matrix alignment. Transcriptome analysis, combined with chromatin biomarkers, revealed these implanted cells upregulated tissue regeneration and wound healing pathways. Collectively our results provide a novel, nongenetic, partial reprogramming of aged cells for cell‐based therapies in regenerative medicine.

## Graphical abstract

Implanted partially reprogrammed cells derived from aged dermal fibroblast show rejuvenation and wound‐healing properties in aged skin model.

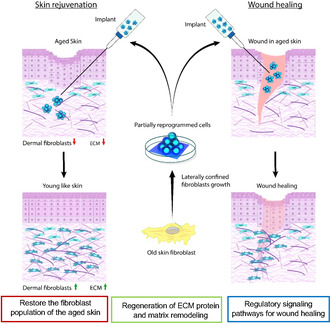

**Abbreviations**
ANOVA: Analysis of variance
AR: Aspect ratio
AU: Arbitrary unit
cDNA: Complementary DNA
DBSCAN: Density‐based spatial clustering of applications with noise
ECM: Extracellular matrix
FT: Full thickness young skin model
FT AGED: Full thickness aged skin model
FDR: False discovery rate
GO: Gene ontology
iPSC: Induced pluripotent stem cell
IPD: Internuclear pairwise distances
LDA: Linear discriminant analysis
MEM: Minimum essential media
MMPs: Matrix metalloproteinases
NA: Numerical aperture
NIH: National Institute of Health; USA
OCT: Optimal cutting temperature
PR: Partially reprogrammed
PDMS: Polydimethylsiloxane
PCA: Principal component analysis
qRT‐PCR: Quantitative real‐time polymerase chain reaction
RNA‐Seq: RNA sequencing
SD: Standard deviation
TPM: Transcripts per millio
Tukey's HSD: Tukey's honestly significant difference
3D: Three dimensional
_μ_m: Micrometre

## 1 INTRODUCTION

Aging skin tissues experience a reduction in dermal fibroblast numbers and an alteration in extra cellular matrix (ECM) protein deposition, resulting in impaired tissue homeostasis and wound healing (Ding et al., 2021; Fisher et al., 1996, 2016; Gurtner et al., 2008; López‐Otín et al., 2013; Wlaschek et al., 2021). In this context, cell‐based therapies including either autologous or allogenic cell transplants have been used to improve tissue repair (Abdel‐Sayed et al., 2019; Bashor et al., 2022; De Buys Roessingh et al., 2006; Dupont et al., 1994; Sun et al., 2014). Autologous implantation involves the implantation of cells derived from the patient's own tissues, while allogenic cell therapies derive cellular implants from healthy donors. However, the major limitations of both therapies include difficulties in harvesting an abundant number of high‐quality cells (e.g., the harvested cells becoming senescent), as well as the complex clinical procedures necessary for isolation and reinfusion (Uchida et al., 1999; Wlaschek et al., 2021). In addition, allogenic transplants have further challenges including immunological rejection and elimination (Petrus‐Reurer et al., 2021; Yu et al., 2019). To overcome this, landmark experiments using cellular reprogramming by Yamanaka factors, namely induced pluripotent stem cell (iPSC) therapies, have become popular for aging tissue regeneration and wound‐healing clearance (Eisenstein, 2022; Roux et al., 2022; Takahashi & Yamanaka, 2006). Reprogramming of aging cells results in epigenetic erasure of aging hallmarks and thus implanting iPSCs offers unique opportunities for tissue regeneration (Bashor et al., 2022; Lu et al., 2020). However, recent experiments have revealed that such iPSC implantation in tissues elicits oncogenic transformation, which limits their clinical applications (Abad et al., 2013; Ohnishi et al., 2014). Although many recent studies are aimed at using partial reprogramming to overcome the clinical bottlenecks of cell‐based therapies (Gill et al., 2022; Ocampo et al., 2016), given the importance of tissue regeneration in personalized medicine, there is a need for alternative approaches, such as nongenetic cell reprogramming‐based therapies.

In recent work, we showed that mechanically confined growth of fibroblasts resulted in their partial reprogramming (Roy et al., 2018). Importantly, such confinement of fibroblasts on micropatterned substrates induced partial reprogramming with high efficiency in the absence of any exogenous reprogramming (Yamanaka) factors. In addition, subsequent redifferentiation of these partially reprogrammed fibroblast—by embedding them in three‐dimensional (3D) collagen‐I matrices with appropriate densities—resulted in their rejuvenation (Roy et al., 2020). This rejuvenation is characterized by increased extracellular matrix protein synthesis and remodeling, and reduced DNA damage compared to the parental fibroblasts. Therefore, we hypothesize that the rejuvenation of aged fibroblasts through partial reprogramming by lateral confinement and subsequent redifferentiation in collagen matrices could provide novel applications for cell‐based therapies.

In this paper, we demonstrate the application of implanting partially reprogrammed (PR) primary human dermal fibroblasts derived from aged individuals as a robust method for tissue regeneration and wound healing. Toward this, we implant mechanically reprogrammed aged fibroblasts into in vitro, reconstructed aged skin tissue models (Bell et al., 1983; Mewes et al., 2007; Nakamura et al., 2018). We assess the degree of in vitro tissue regeneration by quantifying the ECM protein synthesis, remodeling, and the spatial distribution of implanted reprogrammed fibroblasts compared to implanted aged fibroblasts. Immunofluorescence and qPCR assays showed increased expression of collagen, elastin, and fibronectin upon implantation of reprogrammed fibroblasts. Further, we also test the wound healing ability of these PR cells by implanting them into wound sites generated in in vitro aged skin models. To evaluate the wound‐healing efficiency, we quantify ECM protein synthesis as well as their alignment at wound sites. The nuclear and chromatin morphometrics of the implanted PR cells revealed enhanced rejuvenation, and their spatial clustering significantly correlates with their ECM remodeling abilities. Finally, using RNA sequencing of the rejuvenated cells in the in vitro aged skin model, we identified key upregulated genes and signaling pathways involved in the wound healing process. Taken together we demonstrate a nongenetic approach for tissue repair, which may have important implications in regenerative medicine.

## 2 METHODS

### 2.1 Partial reprogramming of aged skin fibroblasts

Skin fibroblasts are procured from the Coriell Institute for Medical Research, USA. Both primary cells GM‐09503 and GM‐08401 were isolated from skin biopsies (lower left abdomen) of healthy persons. The human primary skin fibroblasts were obtained from an aged donor (age 75, GM08401) and a young donor (age 11, GM09503). GM‐09503 cell depository details: GEO Accession No: GSM217865; dbSNP ID: 11267. GM‐08401 cell depository details: dbSNP ID: 15769. All of the primary cells used in this study were from passage 3 to 10. Old cells and young cells were cultured in a minimal essential medium (MEM, Gibco) supplemented with 15% (vol/vol) heat‐inactivated fetal bovine serum (Gibco), 1% nonessential amino acids and 1% penicillin–streptomycin (Gibco). The partially reprogrammed cells derived from old cells were obtained in similar conditions as described before (Roy et al., 2018). Briefly, old cells were grown on laterally confined conditions on a specific fibronectin micropattern (area 9000 μm^2^ with AR 1:4) created on the surface of uncoated culture dishes (catalog no. 81151, Ibidi) by stamping fibronectin‐coated (catalog no. F1141, Sigma) polydimethylsiloxane (PDMS) micropillars fabricated by soft lithography. Single fibroblasts grown on these patterns under laterally confined conditions for 8 days in the abovementioned culture medium resulted in partially reprogrammed spheroids. These spheroids exhibited stem‐like properties as previously characterized and named as partially reprogrammed (PR) cells (Roy et al., 2020). Each batch of these PR cells were characterized by alkaline phosphatase assay prior to their further application. After 8 days, these spheroids of the PR cells were extracted from the culture plate by scraping and collected in a small volume of culture media. The young and old fibroblasts were used for respective control conditions. In control conditions (old and young), similar spheroid size and cell density (compared to PR spheroids) was achieved by seeding corresponding cells on micropattern dishes (500 μm) at a concentration of ∼80,000 cells per dish and growing them overnight. These spheroids were also extracted as described above.

### 2.2 In vitro skin models, wound formation, and cell implantation

In this study, we used two types of reconstructed, full thickness (FT) skin models (Phenion™). The FT AGED skin model is a surrogate for aged human skin and was used as an aged skin model in our study. The FT AGED skin model is characterized by connective tissue with senescent fibroblasts, reduced synthesis of ECM proteins like collagen and elastin, and elevated matrix metalloproteinases (MMPs) secretion. The FT Standard model with standard ECM and fibroblast constituents in the dermis and epidermis was used as a young skin model. To initiate the wound, we used the following procedure. Initially, we demarcated the wound area using a 1.5‐mm biopsy punch. Subsequently, a scalpel was employed to excise a roughly 2‐mm deep V‐shaped skin section, encompassing both the epidermis and dermis. This excisional biopsy method was executed as illustrated in Figure S7. Distinct spheroids of various cell types (PR, old, or young) were subsequently implanted into separate in vitro skin models, namely FT, FT AGED, or FT AGED wound models. For each implantation, spheroids equivalent to 40,000 cells were resuspended in 50 μL of growth media. Implantation was carried out with a 1 mL syringe and a 25‐gauge needle. In every skin model, 2–3 implantation regions consisting of the same type of cells were established. In the context of skin rejuvenation experiments, cells were implanted within the 2‐mm‐deep dermal region. However, for wound‐healing models, cells were implanted both at the wall and tip regions of the V‐shaped wound structure. This strategic placement of cells is intended to assess their impact on wound‐healing dynamics within these distinct regions.

### 2.3 Cryo‐sectioning and immunofluorescence of tissue sections

After 10 days of culture, the tissues are cryopreserved according to the manufacturer's protocol. Briefly, the skin model was first dissected into two nearly identical halves, and then the tissue halves were again cut parallel to the first section plane. The dissected tissues were placed in a precooled cryo‐tray and were frozen using OCT cryo embeded medium (OCT, Leica Biosystem). After the tissue‐freezing medium was frozen completely, the skin model pieces were stacked side‐by‐side with the larger cutting edge facing upward and leaving a small space between each piece. Fresh tissue‐freezing medium was added slowly around the tissue and wait for freezing and repeated the step until the tissues were completely embedded in the frozen medium. The frozen tissues were cryosectioned at 7 μm thickness using a cryomicrotome (Leica) and transferred to Superfrost Plus slides (Thermo Scientific). Tissue sections were dried, cut, and stored at −80°C until further use. For immunostaining, slides were recovered from −80°C, dried briefly, and fixed in −20°C (ice cold) acetone for 15 min. Then slides were dried for 5 min, and tissue sections were encircled with a PAP pen (Sigma‐Aldrich) and dipped in phosphate buffer saline (PBS) for a few minutes. All subsequent steps were performed in a humid chamber. Tissue sections were blocked in 10% goat serum for 1 h. The slides were incubated with primary antibody solutions (Table S1) diluted in 1% bovine serum albumin (BSA) and 0.3% Triton in PBS at 4°C overnight. Then, slides were washed in PBS three times for 5 min each. Slides were incubated with Alexa Fluor plus secondary antibodies (Table S1) prepared in 1% BSA and 0.3% Triton. Again, slides were washed with PBS three times. After the wash, samples were stained with Hoechst 33342 (NucBlue, Thermo Fisher Scientific) in PBS for 10 min at room temperature, and further mounted with mounting media (Gold antifade, Thermo Fisher Scientific) and a coverslip. Slides were left to dry overnight at room temperature and stored at 4°C until imaging.

### 2.4 Image acquisition and image analysis

Immunofluorescence images of tissue sections were acquired by using a Nikon A1R laser‐scanning confocal microscope (Nikon Instruments Inc.) at either 20× magnification (Plan Apo 20× extra‐long working distance, numerical aperture [NA] 0.8) or 40× magnification (1.25‐NA silicon objective) with identical acquisition settings and a step size of 3 and 1 μm, respectively. Confocal images of either 512 × 512 or 1024 × 1024 pixels were obtained with an XY optical resolution of 0.84 or 0.42 μm, respectively.

#### 2.4.1 Intensity analysis

The fluorescence intensity of the cells at the implanted region was measured for each protein in its respective channel using custom‐written code in Fiji (NIH). We computed the fold change of the ECM protein levels and quantified the normalized mean intensities of the PR cell‐implanted regions (with respect to the mean normalized intensity of the old cell implanted regions).

#### 2.4.2 Internuclear pairwise distance (IPD) analysis

The internuclear pairwise physical distances (IPD) for the implanted cells were computed from the nuclear positions in the skin model, as reported by Iyer et al. (2012). Nuclear positions were extracted from the coordinates of centroids of the nuclei obtained from images of Hoechst 33342 stained tissue sections. Internuclear pairwise physical distances were computed as:

$$\mathrm{IP}D_{\mathrm{ij}}=<\left|r_{i}-r_{j}>\right|=\left\langle \sqrt{{\left(x_{i}-x_{j}\right)}^{2}+{\left(y_{i}-y_{j}\right)}^{2}}\right\rangle ,$$

where *r*_*i*_ and *r*_*j*_ are the physical distances between the nuclei centroids. IPD matrices were constructed by the mean distances between centroid positions of the nuclei in which the (*i*, *j*)th element represents the internuclear physical distance between *i*th and *j*th nuclei in the implanted region.

#### 2.4.3 ECM alignment analysis

The fiber alignment of collagen I deposited by the implanted cells was computed by the OrientationJ Fiji plugins (Püspöki et al., 2016). The collagen I fiber alignment at the wound site during wound healing was computed from the images of collagen I stained immunofluorescence sections. With a user‐defined size of a Gaussian‐shaped window, the program computes the structure tensor for each pixel in the image by sliding the Gaussian analysis window over the entire image. The local orientation properties with respect to the *x*‐axis of the images were computed and were then visualized as color images with the orientation being typically encoded in the color (hue).

#### 2.4.4 Nuclear feature analysis

From the original Hoechst 33342 images, local and global Otsu thresholding was used to segment the nucleus. The large and dim autofluorescent regions were removed based on their size and fluorescent intensity. The nuclear features were then extracted using our previously reported method (Venkatachalapathy et al., 2021), https://github.com/GVS‐Lab/chrometrics). Based on these nuclear features, linear discriminant analysis (LDA) was used to train a classifier for distinguishing injected old and PR cells. For training with the balanced dataset, 3000–3500 nuclei were used from each condition. 75% of the nuclei were used for training and the remaining 25% were used for testing. The LDA1 values were normalized to a range of 0–1 to derive a score for each nucleus. For analyzing the spatial distribution of the injected cells based on their nuclear features, we used density‐based spatial clustering of applications with noise (DBSCAN) of the nuclear features and centroid coordinates within the image. All of these features were normalized to a range of 0–1 before the clustering analysis. Python hdbscan was used in this study (McInnes et al., 2017).

### 2.5 RNA‐Seq sample preparation and analysis

Total RNA was isolated from the implanted cells specifically in the wound regions. After implantation and 10 days of culture, the FT AGED wound models were precisely dissected to collect the tissue of the wound site and surrounding tissue regions. The epidermis of the dissected tissue was removed, and the remaining sections were minced finely for further processing. The total RNA was isolated according to the manufacturer's protocol using the RNeasy Mini Kit (Qiagen; Hilden, Germany). The preparation of the mRNA library (Illumina's TruSeq Stranded protocol) and sequencing on a HiSeq 2000 platform were performed at the Department of Biosystems Science and Engineering (DBSSE), ETH Zurich. In summary, we had two conditions: PR (implanted PR cells in AG tissue‐based wound model) and old (old fibroblasts implanted in the FT AGED wound model). Each condition had two biological replicates and technical replicates (run on four different lanes). RNA analysis was done as described previously (Roy et al., 2020). Paired end reads were aligned to Homo sapiens GRCh38.84 reference genomic indexes using the HISAT2 sequence‐alignment tool (version 2.2.1). The cloud indexes (grch38_trans) for HISAT2 were accessed on 25 June 2020 from https://registry.opendata.aws/jhu‐indexes. Four technical replicates for each biological sample were combined as input of HISAT2. Default parameters were used in HISAT2. Single aligned reads were counted by htseq‐count (1.99.2). Count numbers for all expressed genes were used in differential expression analysis using DESeq2 (Version 1.34.0). Differentially expressed genes had adjusted *p* values (Benjamini–Hochberg) below a 0.1 false discovery rate (FDR) and fold change higher than 2. Enrichment analysis was done with the DAVID database (December 2021 version). Gene lists for each Gene Ontology (GO) term were obtained from AmiGO. Genes used in the principal component analysis (PCA) are from GO:0009611, GO:0030198, and GO:0042060. For each gene, a z‐score value measured in transcripts per million (TPM) from one set of experiments was used. Highly correlated genes (correlation coefficient >0.95 or < −0.95) were removed in this PCA. Principal component (PC) one was chosen as the combined expression level.

### 2.6 qRT‐PCR

To investigate selected gene expressions, qRT‐PCR was performed from the extracted total RNA from both tissue rejuvenation and wound healing models. The total RNA was extracted using the abovementioned procedure. Complementary DNA (cDNA) was synthesized from total RNA using an iScript cDNA synthesis kit (Bio‐Rad). Real‐time PCR detection was performed by using a SsoFast qPCR kit (Bio‐Rad) for 40 cycles in a Bio‐Rad CFX96. The relative fold changes in the gene levels were obtained from qRT‐PCR data, by calculating ΔΔCt with respect to glyceraldehyde 3‐phosphate dehydrogenase (GAPDH) levels. The primer sequences for the selected genes are listed in SI Appendix, Table S2.

### 2.7 Statistical analysis

All data are expressed as mean ± SD and ± SEM as noted in figure legends. For box plots, the box limit represents the 25–75th percentile and whiskers 1.5× interquartile range. Each experiment was repeated at least three times. We evaluated the statistical significance of the different set of data with the one‐way ANOVA, Tukey's HSD test, Mann–Whitney *U* test and Student's paired two‐tailed *t* test as applied. *p < 0.05; **p < 0.01; ***p < 0.001.

## 3 RESULTS

### 3.1 Implanted, partially reprogrammed spheroids show rejuvenation properties in an FT AGED skin model

**FIGURE 1:**
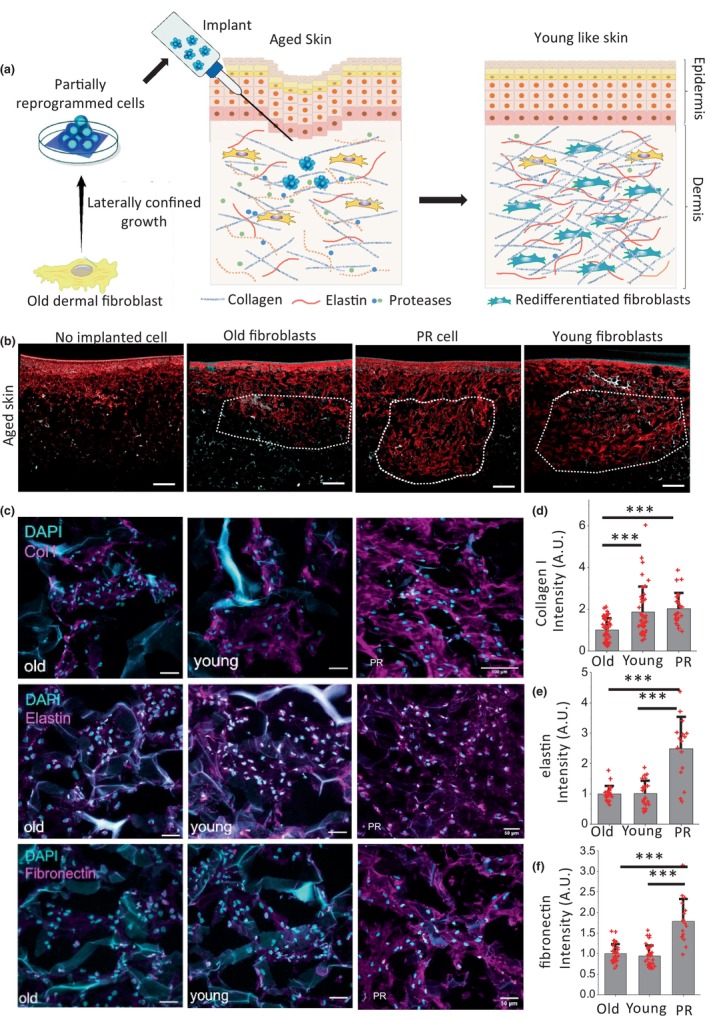
Implanted PR cells show rejuvenation properties in an aged skin model. (a) Schematic representation of the in vitro skin model for tissue regeneration by mechanically reprogrammed cells. (b) Representative images (10× magnification) of immunofluorescence tissue sections of in vitro, aged skin tissue with different implanted cells immunostained with collagen I antibodies (red) and hoechest (cyan). Scale bar: 500 μm. (c) Representative images (40× magnification) of the cell‐implanted regions of in vitro aged skin tissue sections stained with collagen I, elastin, and fibronectin antibodies. Scale bar: 50 μm. (d–f) Normalized intensity plots of ECM proteins collagen I, elastin and fibronectin at the cell‐implanted regions obtained from the immunofluorescence images of in vitro aged skin tissue sections. Statistical analysis for the quantification of fluorescent intensity (A.U. unit) was performed with three biological replicates. *p* value is from Tukey's HSD test. ***p value < 0.001.

In our previous studies, we showed that primary, aged skin fibroblasts can be rejuvenated through lateral confined growth‐induced mechanical reprogramming when followed by redifferentiation in a 3D collagen matrix (Roy et al., 2018, 2020). Through this rejuvenation approach, we observed that the rejuvenated cells showed a characteristic, young fibroblast‐like phenotype including enhanced cell size, contractility, and ECM remodeling capabilities. Importantly, these young fibroblast‐like phenotypes are essential for the rejuvenation of aged human skin by restoring the fibroblast population, regenerating ECM proteins, and matrix remodeling. To acquire partial reprogrammed (PR) cells, primary old skin fibroblasts obtained from an aged donor (age 75, GM08401, Coriell Institute) were grown in laterally confined conditions on a fibronectin micropattern (area 9000 μm^2^ with aspect ratio [AR] 1:4) for 8 days as characterized in Figure S1. To explore the rejuvenation potential of these PR cells in human skin, here we implanted these PR cells in the dermal area of the reconstructed FT AGED skin model (Phenion™) and allowed them to differentiate and grow for 10 days (Figure 1a). The skin model mimics mature human skin, both in structure and physiology (Malhotra et al., 2019; Mewes et al., 2017). It is characterized by connective tissue with senescent dermal fibroblasts, reduced synthesis of ECM proteins like collagen I and elastin, and elevated MMP secretion. After implantation, these skin tissues were cultured for 10 days in air‐liquid culture conditions, followed by cryo‐embedding and cryo‐sectioning for further immunofluorescence staining. To compare the ECM protein regeneration efficiency of different types of cells, we separately implanted old fibroblasts, young fibroblasts and PR cells in aged skin tissues and evaluated their protein production by immunofluorescence staining for collagen I. Interestingly, we observed that the implanted PR cells synthesized collagen I at similar levels as young cells, but produced much higher collagen I than the implanted old cells, as represented in the large field images of immunofluorescence tissue sections of the in vitro, aged skin tissue stained with collagen I antibody (Figures 1b and S2). Further, we showed that the regeneration of other important ECM proteins including elastin and fibronectin were also efficiently produced by these implanted PR cells (Figures 1c and S3). The quantitative image analysis clearly showed the level of collagen I, elastin and fibronectin in the FT AGED skin model produced by the implanted PR cells was significantly higher than the implanted old fibroblasts (Figure 1d–f). Unlike the PR cells, the implanted young cells were only able to regenerate significantly more collagen I, but not elastin and fibronectin, in the aged skin compared to implanted old cells. Further, in agreement with the immunofluorescence data, the increase in messenger RNA (mRNA) levels of selected ECM‐related genes was validated by qPCR assay (Figure S4). In addition, we also tested the ECM regeneration ability in young tissue by these PR cells. Similarly, we implanted these three types of cells in the in vitro, full thickness standard skin (young skin). By immunofluorescence analysis we observed that PR cells regenerated significantly more collagen I and fibronectin in FT skin model compared to young and old cells (Figure S5). We also observed that elastin regeneration in young tissue by these three types of cells was not significantly different. Collectively, these results suggest that PR cells, upon implantation in an FT AGED skin model, efficiently regenerate ECM proteins to rejuvenate the aged skin into young‐like skin.

### 3.2 Implanted PR cells redifferentiate to fibroblast‐like characteristics with enhanced spreading efficiency in the FT AGED skin model

**FIGURE 2:**
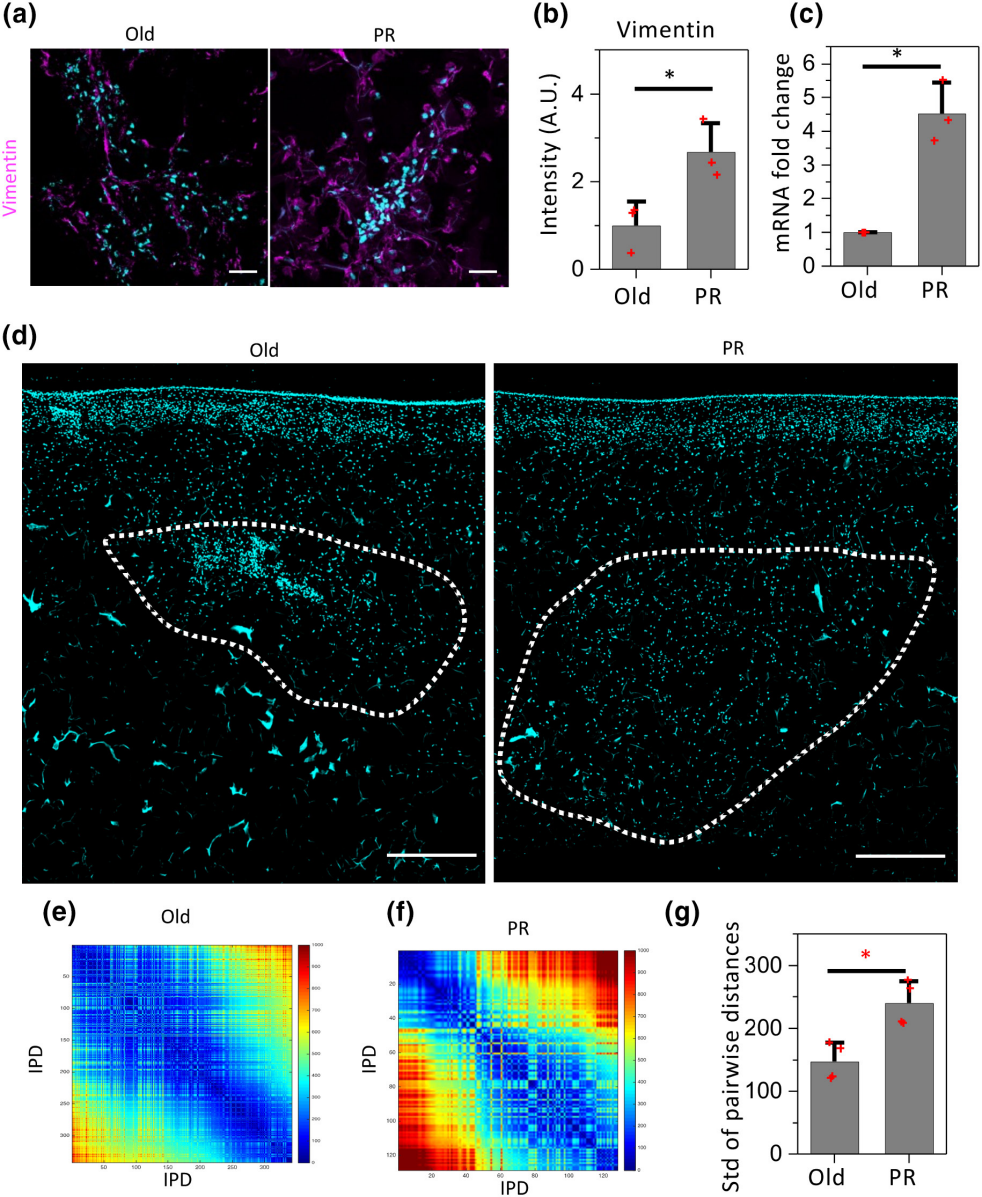
Implanted PR cells show fibroblast properties with enhanced spreading area in the aged skin model. (a) Representative immunofluorescence images of the in vitro aged skin tissue sections stained with vimentin antibodies. Nuclei are stained with hoechest (cyan). Scale bar: 50 μm. (b) Normalized intensity plots of vimentin proteins in the implanted cells obtained from immunofluorescence tissue sections. *p* value is from Mann–Whitney *U* test. *p value <0.05. (c) Bar plots representing the mRNA levels of fibroblast specific gene vimentin (Vim) in the implanted PR cells obtained by qRT‐PCR normalized with respect to implanted old cells. Statistical analysis for the qPCR experiments was performed using a paired Student *t* test. *p value < 0.05. (d) Representative nuclear images of the implanted old and PR cells in immunofluorescence tissue sections stained with hoechest (cyan). Scale bar: 500 μm. (e,f) Representative inter‐physical distance (IPD) matrix of the implanted cell nuclei in immunofluorescence tissue sections. Color code: warmer colors represent larger inter‐chromosome distances and cooler colors represent smaller distances. Rows and columns indicate nuclear pairwise distances in μm. (g) Box plot representing the standard deviations of nuclear pairwise distances in implanted old and PR cells. The *p* value is obtained from a Mann–Whitney *U* test. *p value < 0.05.

To characterize the functional state of the implanted cells in the in vitro aged skin, we tested the fibroblast‐related marker vimentin. Through immunofluorescence staining of tissue sections, we observed that the implanted PR cells expressed comparatively higher vimentin than the implanted old cells (Figure 2a,b). In agreement with this, the mRNA levels of the corresponding genes extracted from the implanted region of the skin tissue also showed a similar trend (Figure 2c). These results suggest that upon implantation in the in vitro skin tissue matrix, the stem cell‐like PR cells redifferentiate with fibroblast characteristics, but do not display a fibrosis signature, which is useful for tissue rejuvenation therapies. For efficient rejuvenation it is essential that the implanted cells distribute to the appropriate area of the skin to maintain proper local cell density and effective matrix regeneration. To characterize the spreading efficiency of PR cells upon implantation in the FT AGED skin model, we computed internuclear pairwise physical distances (IPD), as described by Iyer et al. (2012). Hoechst‐stained images of immunofluorescence tissue sections were used to map nuclear positions (Figure 2d). IPD matrices were constructed by the pairwise distances of the nuclear centroids from the implanted region (Figure S6a). IPD matrices are symmetric with diagonal elements of zero. We observed that the IPD values of the PR cells were higher than the old cells' (Figures 2e,f, and S6b). Further, to quantify the spatial distribution of the implanted cells across repeat experiments, we calculated the standard deviation of the IPDs in these two conditions. In agreement with the IPD matrices, we observed that standard deviations of the IPDs in PR cells are significantly higher than the old cells (Figure 2g). These results clearly highlight that implanted PR cells uniformly spread into a larger area of the in vitro aged skin tissue for effective ECM regeneration compared to the implanted old fibroblast cells.

### 3.3 Enhanced ECM regeneration and proper matrix alignment by the implanted PR cells facilitate wound healing in an FT AGED skin wound model

**FIGURE 3:**
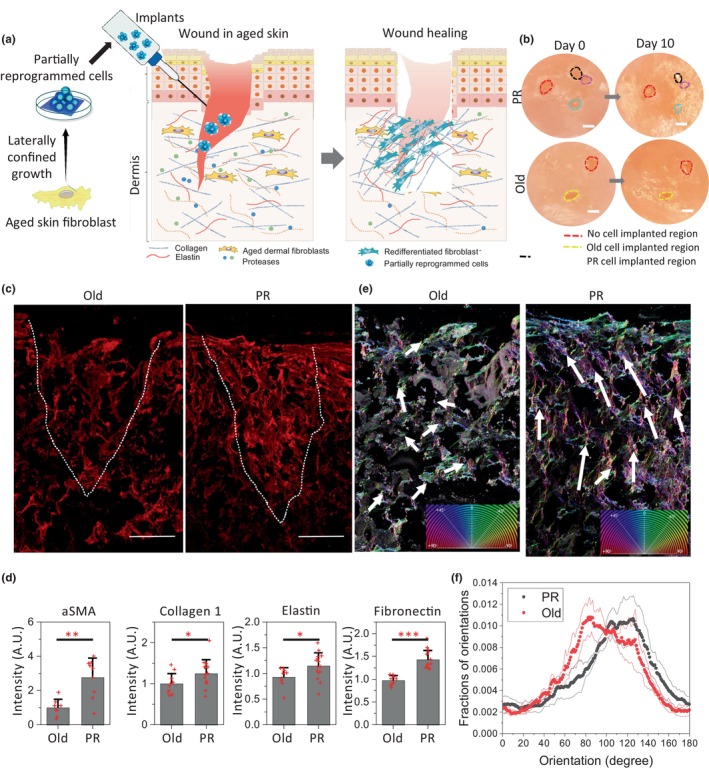
ECM production by the PR cells facilitates wound healing in the aged tissue model. (a) Schematic representation of the in vitro aged skin model for wound healing by mechanically reprogrammed cells. (b) Representative top surface view of the wounds on the in vitro aged skin models before implantation and 10 days after implantation. (c) Representative images of immunofluorescence tissue sections of wounds with implanted cells. Matrix stained with collagen I (red). Scale bar: 500 μm. (d) Normalized intensity plots of αSMA proteins in the implanted cells and ECM proteins collagen I, elastin and fibronectin at the implanted wound regions. Statistical analysis for all graphs were performed with two biological replicates. *p* value is from Mann–Whitney *U* test. *p value < 0.05, **p value < 0.01, ***p value < 0.001. (e) Representative input fluorescence images of collagen I fibers at implanted wound regions obtained from the tissue sections. Reconstructed HSB color‐coded map of the collagen I fibers, hue: angle of the local orientation, saturation: coherency, brightness: input image. Small images in the right bottom corners, the color representation reflects the different orientations. (f) Histogram of the local orientation of collagen I fibers at the implanted wound site during wound healing.

To test the synthesis of ECM proteins and their matrix remodeling properties in the wound‐healing process, we implanted PR cells in an FT AGED skin wound model. The wound model was obtained by making a sharp, deep cut in the FT AGED skin tissue model (Figures 3a and S7). PR cells were implanted in the wound site and nearby regions of the skin and followed by immunofluorescence staining of tissue sections after 10 days. The marked regions of the images from the top surfaces of the aged skin showed the wound region on Day 0 and Day 10 after implanting cells. From these top view images, we found that the wound with implanted PR cells showed better wound closure than the wound with implanted old cells (marked yellow arrowheads in Figure 3b). However, the wound regions without implanted cells that are marked in blue arrowheads did not show significant changes even after 10 days. By immunofluorescence staining of tissue sections, we found that the implanted cells regenerate the ECM at the wound site, whereas there was no ECM regeneration in the non‐implanted condition (Figures 3c and S8). Like the tissue rejuvenation model, in the wound site we also observed significantly higher secretion of ECM proteins including collagen I, elastin and fibronectin by the PR cells compared to old cells (Figures 3d, S9 and S10). This enhanced ability of ECM regeneration of PR cells at the wound site facilitates the wound healing process. As alpha smooth muscle actin (αSMA) has been suggested to play an important role in the production of contractile force during wound healing (Darby et al., 1990; McAndrews et al., 2022; Tomasek et al., 2002), we tested the expression level of αSMA at the wound site. Interestingly, the implanted PR cells at the wound site showed enhanced production of αSMA (Figures 3d and S11). We suggest that the enhanced expression of collagen I and αSMA, markers of fibroblast activation, results in the wound‐healing process (Higashiyama et al., 2011). In contrast, we did not observe upregulation of αSMA in the tissue rejuvenation model. This signifies that the implanted PR cells are sensitive to the wound microenvironment (Figure S11). In addition to ECM synthesis, the alignment of the ECM fibers is a crucial precursor for collective cell migration in wound closure (Kubow et al., 2015; Martin & Lewis, 1992; Pfisterer et al., 2021; Reinke & Sorg, 2012; Sharma et al., 2017). To analyze the alignment of the newly synthesized ECM, we quantified the orientation of the collagen I fibers within and nearby regions of the wound site using OrientationJ (Püspöki et al., 2016) image analysis tools (Figure S12). From the orientation analysis we found that the newly synthesized collagen I fibers mediated by PR cell implantation were more aligned with respect to the wound site compared to the random fiber organization observed to be produced by the implanted old cells (arrowheads in Figure 3c and HSB colour map in Figure 3e). Moreover, the width of distributions of the fiber orientations with PR cells are smaller compared to the old cells (Figure 3f). Collectively, the above results highlight the efficient ECM remodeling and wound‐healing ability of the PR cells upon implantation into the wound microenvironment.

### 3.4 Implanted PR cells exhibit different nuclear and chromatin features from old cells in both skin rejuvenation and wound‐healing models

**FIGURE 4:**
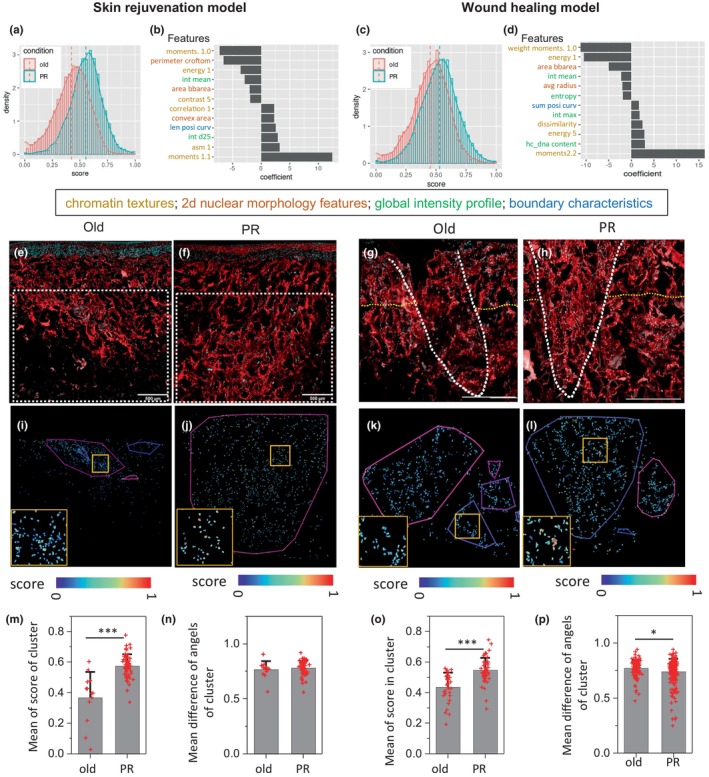
Implanted PR cells exhibit specific nuclear and chromatin features from old cells in both skin rejuvenation and wound‐healing models. (a,c) Probability density histograms of the normalized LDA1 score for nuclei from injected old (red) or PR (blue) cells. (b,d) Top nuclear features used in the LDA classifier and the related coefficients. (e–h) Representative images of the imaged regions in the skin rejuvenation and wound healing models. (i–l) The corresponding spatial clustering of nuclei based on their LDA scores from the same region. (m–o) Barplot shows the mean of the LDA scores within the local clusters for the injected old and PR cells. (n,p) Barplot shows the mean of the angular difference between any two nuclei within the cluster for the injected old and PR cells. *Student *t* test *p* value < 0.5;***Student *t*test *p* value < 0.001.

After injecting PR cells into the two skin tissue models, it is important to check the cellular states and spatial distribution of each state in the skin tissue during the rejuvenation and wound healing processes. Previous work from our group showed that nuclear morphology and chromatin organization features could be used as a biomarker of cell states, and any changes in cell state could be classified using the nuclear features (Venkatachalapathy et al., 2021). To quantify single‐cell chromatin features, individual nuclei were segmented using Otsu thresholding and four groups of nuclear and chromatin features (chromatin texture, nuclear morphology, global intensity profile and nuclear boundary characteristics) were extracted from them. Based on the LDA, we found that the linear combination of these four groups of features could distinguish injected PR cells from old cells with high accuracy (~63%–67% for testing) for both the rejuvenation and wound‐healing processes (Figure 4a,c). For the rejuvenation process, chromatin textures and 2D nuclear morphology features are important for classifying the cell states (Figure 4b). Alternatively, the wound healing process was accompanied by alterations in the chromatin texture and global intensity profiles (Figure 4d). To study the spatial distribution of these injected cells in these two different models (Figure 4e–h), we used the DBSCAN (Ester et al., 1996) method to group nuclei with similar LDA scores (Figure 4i–l). We found that the injected PR cells from the same local groups have significantly higher LDA scores compared to the old cell groups for both rejuvenated and wound‐healing models (Figure 4m,o). This indicates that injected PR cells locally are maintained at different cell states from the old cells, which confirms the previous observations that contractility and ECM secretion are different between these two types of injected cells. Besides, we also found that the angular differences within the local clusters of injected PR cells were smaller than the old cell clusters in the wound‐healing model but not in the rejuvenation model (Figure 4n,p). This suggests that these locally aligned, injected PR cells may contribute to their enhanced wound‐healing ability.

### 3.5 Implanted PR cells at wound sites acquire different transcription profiles with enhanced ECM and wound‐healing gene expression

**FIGURE 5:**
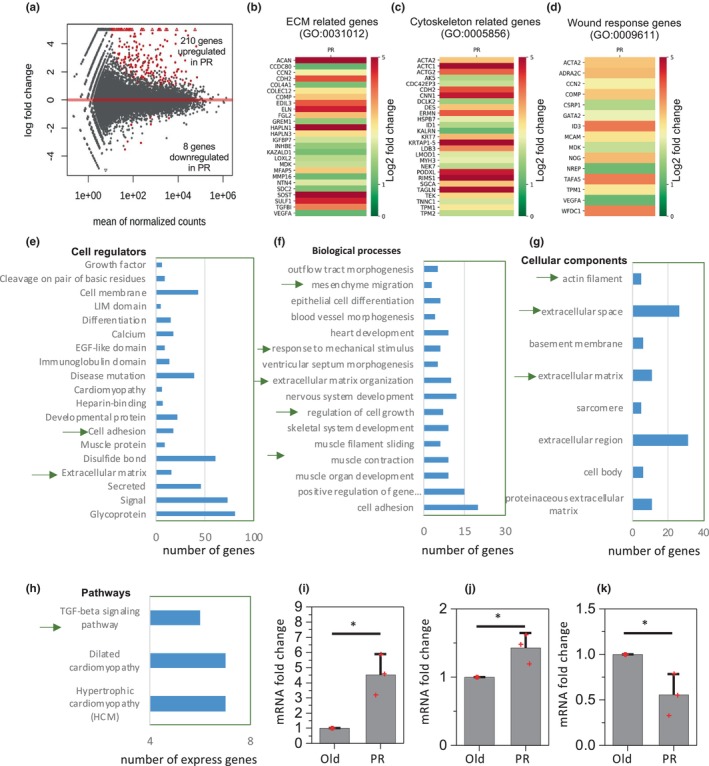
RNAseq analysis and qPCR validation. (a) MA‐plot for the log2 fold changes shows the differentially expressed genes marked in red. The significant differentially expressed genes have adjusted *p* values < 0.1 (Independent Hypothesis Weighting implemented in DESeq2) and fold change >2. (b–d) Heatmaps show the log2 fold changes of the upregulated genes in injected PR cells compared to the injected old cells in three GO annotated gene lists. GO:0031012 refers to the cellular component extracellular matrix, GO:0005856 refers to the cellular component cytoskeleton, and GO:0009611 refers to the biological process response to wounding. (e–h) Barplot shows the number of significant differentially expressed genes annotated by each significant enriched term from the selected categories. For this enrichment analysis, significant enriched terms have the Benjamini *p* value < 0.1. (i–k) qPCR experiments show the change of ACTA2, COL1a1, and MMP9 comparing injected old and PR cells. Statistical analysis for the qPCR experiments was performed using a paired Student *t* test. *p value < 0.05.

To further characterize the gene expression profile during the abovementioned wound‐healing process, we extracted the RNA from the wound site after 10 days of implantation and performed global RNA sequencing. From RNA‐Seq analysis we observed that more than 200 genes (210 genes) were significantly upregulated (fold change >2, adjusted *p* value < 0.1) and 8 genes were downregulated in the PR cells with respect to the old condition (Figure 5a). Interestingly, among most of the upregulated genes in PR cells, we found three groups of genes were predominantly upregulated, namely ECM‐related genes (GO:0031012), cytoskeleton‐related genes (GO:0005856), and wound response genes (GO:0009611) (Figure 5b–d). In addition to the proteins observed in the immunofluorescence images of tissue sections, through this GO analysis, we also identified other upregulated genes including Acan, Col4a1, Loxl2, Mmps, and Tgfb1 that have important roles in ECM biosynthesis and remodeling (Figure 5b). In addition, we also observed that several cytoskeletal‐related genes, including Acta1/2, Cdh2, Ermn, and Krt7, were upregulated which potentially facilitates matrix contraction during wound healing (Figure 5c). Further, we observed several other genes were upregulated that have an important role in the wound‐healing process (Figure 5d). This upregulation of ECM biosynthesis and remodeling, cytoskeletal contractility and cytoskeletal regulation are further confirmed by the functional enrichment analysis of the differentially expressed genes (Figure 5e–g). Moreover, in this PR condition we also found the upregulation of transforming growth factor beta (TGF‐β) pathways, which is a well‐known regulator of wound healing (Figure 5h). Through principal component analysis of RNA‐Seq data from various stages of in vivo wound‐healing processes, available from the literature, we found that our PR cell‐implanted wound model showed a similar trend of combined expression change in the in vivo wound‐healing process (Figure S13). Further, in agreement with the RNAseq, the increase in mRNA levels of selected ECM and contractility‐related genes was validated by qPCR (Figure 5i–k). These results show that upon implantation into the wound site, PR cells can redifferentiate into a fibroblast‐like state and show molecular upregulation of ECM, contractility, and wound‐healing pathways for faster and more efficient wound healing.

## 4 DISCUSSION

In summary, our study demonstrates the potential of implanting mechanically reprogrammed aged fibroblasts for tissue regeneration and wound healing. We utilized an in vitro FT skin model that mimics physiologically relevant young and aged skin tissue. As expected, implanting only aged fibroblasts resulted in minimal ECM regeneration and remodeling. However, when the PR cells were implanted, they underwent redifferentiation, leading to a highly rejuvenated fibroblastic cell state. This rejuvenation was evident through the expression of fibroblast markers like vimentin and the deposition of increased ECM, including collagen I, elastin, and fibronectin. Furthermore, the rejuvenated cells demonstrated enhanced migratory capacity compared to aged fibroblasts, indicating their tissue regenerative potential over larger distances. Our results suggest that implanting PR cells in aged tissues could offer highly regenerative outcomes with both increased cell numbers and spatial distributions.

In our investigation, we also compared the regenerative efficacy of implanting rejuvenated fibroblasts derived from 3D collagen matrix‐mediated redifferentiation with direct implantation of PR cells (without redifferentiation) into the in vitro, reconstructed FT skin model. The results indicated that direct implantation of PR cells lead to higher ECM regeneration and remodeling, which holds promise for cell therapy models targeting skin rejuvenation.

Furthermore, aging also leads to the loss of wound‐healing properties in the skin tissue due to reduced fibroblast numbers in the dermis and decreased ECM regeneration and remodeling properties (Ding et al., 2021; Fisher et al., 1996, 2016; Gurtner et al., 2008; López‐Otín et al., 2013; Wlaschek et al., 2021). To address this, we explored whether implanting the PR cells derived from aged fibroblasts could serve as novel cell‐based therapies for wound healing. In aged skin tissue with sharp wounds, we compared the effects of implanting old fibroblasts and PR cells derived from the same fibroblasts. Additionally, for a skin rejuvenation approach, we compared the outcomes of implanting young, old and PR cells in both FT and FT AGED skin models. However, as slow wound healing is more relevant to old skin than to young skin, and the implantation of PR cells derived from autologous old cells is clinically valuable, we only compared the efficiency of wound healing between PR cells and old cells. Notably, PR cells exhibited enhanced ECM protein generation at the wound site and aligned local ECM fibers, facilitating an accelerated wound healing process. Imaging‐based chromatin features were used to characterize the cell states of the implanted aged or PR cells, further confirming their redifferentiation, regeneration, and wound‐healing properties in both model systems.

To gain better functional insights into wound healing, we carried out RNA sequencing on cells extracted from the wound site. Differential analysis of gene expression showed the upregulation of ECM genes, cytoskeletal genes, and various signaling pathways related to wound healing for PR cells compared to old cells. In particular, we identified a cluster of 20 growth factors, including VEGFA, TGFBI, IGFBP3, IGFBP6, and IGFBP7, exhibiting a significant RNA expression increase in the injected PR cells when compared with the injected old cells. This underscores the pivotal role of growth factor secretion in the process of tissue rejuvenation. Interestingly, MMP16 displayed marked upregulation in the PR cells in comparison to the old cells, although other MMP genes did not display significant differences under the current experimental protocol (Figures S7 and S14). In addition, our GO analysis did not reveal differentially expressed genes linked to extracellular matrix disassembly. Furthermore, cytoskeletal genes such as Acta1/2, N‐Cdh2, Ermin, CTGF, GATA2, ID3, and Midkine were differentially regulated in the injected PR cells. Previous studies have shown that these cytoskeletal genes have important roles in wound healing (Aleksander et al., 2023; Brockschnieder et al., 2006; De Wever et al., 2004; Momtazi‐Borojeni et al., 2018; Rockey et al., 2013; Suresh & Diaz, 2021; Wang et al., 2016).

In the context of in vitro FT skin models, it is essential to acknowledge that while they capture some aspects of aged skin tissue, they lack the full complexity of human skin with other cell types, including immune cells. Nonetheless, our in vitro model demonstrates the regenerative capabilities of partially reprogrammed aged fibroblasts when implanted in FT AGED skin tissue. Indeed, the partially reprogrammed cells at the wound sites show increased αSMA, vimentin, and TGF‐β signaling pathway activity, which are hallmarks of activated fibroblasts. The activation of fibroblasts and the involvement of immune cells play critical roles in in vivo skin wound healing to maintain tissue homeostasis (Griffin et al., 2021; Mishra et al., 2019; Park & Barbul, 2004). Despite this limitation, our study lays the foundation for understanding the behavior of implanted PR cells and their contribution to tissue regeneration and wound healing.

The deposition of ECM proteins could also suggest activation of fibrotic pathways. To address this, we assessed the duration of PR cell activation in the rejuvenated state. Our findings indicated that after 2–3 cell division cycles, the matrix deposition by PR cells were reduced. Furthermore, we evaluated αSMA expression, a crucial marker of skin fibrosis. Interestingly, αSMA expression was noticeable in the implanted PR cells only in sites of wounds. This aligns with the understanding that fibrotic induction is necessary during the initial stages of wound healing. The αSMA expression suggests that the implanted cells sense and respond to the mechanical microenvironment of the surrounding tissue region. In conclusion, our results reveal exciting possibilities for personalized cell‐based therapies by obtaining patient specific aged cells, reprogramming them in vitro and subsequently implanting them back into the same patient for tissue regeneration and wound‐healing applications.

## AUTHOR CONTRIBUTIONS

Bibhas Roy, Tina Pekec, and G. V. Shivashankar designed research; Bibhas Roy and Tina Pekec performed research; Bibhas Roy, Tina Pekec, Luezhen Yuan, and G. V. Shivashankar analyzed data; and Bibhas Roy, Tina Pekec, and G. V. Shivashankar wrote the paper.

## CONFLICT OF INTEREST STATEMENT

The authors declare no conflict of interests.

## Supporting information


**Data S1:** Supporting information.

## Data Availability

Any additional display items, table and source data are available in the SI Appendix. All relevant data are within the manuscript and its SI Appendix files. The RNA‐seq datasets generated in this study can be found in Dataset S1.
