# Supplementary material for: Implanting mechanically reprogrammed fibroblasts for aged tissue regeneration and wound healing
Source: Aging Cell. 2023 Nov 27;23(2):e14032. doi: 10.1111/acel.14032 (PMC10861198; doi:10.1111/acel.14032)
Supplement: Supplementary file 1 — Data S1: Supporting information. [file ACEL-23-e14032-s001.zip › Revised Supplentary information.pdf]

SUPPLEMENTARY FIGURES

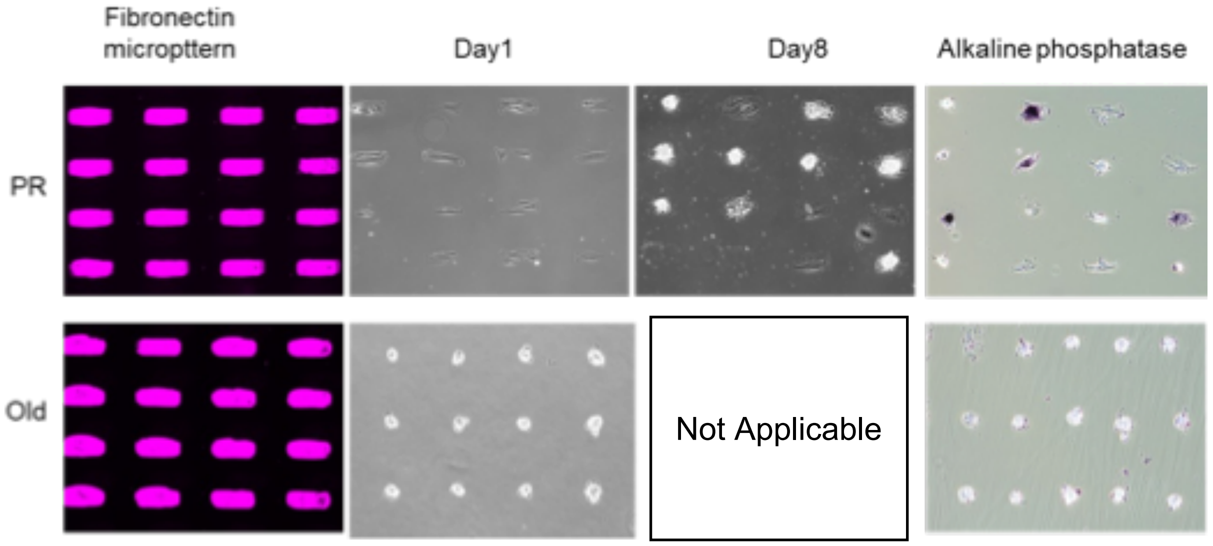

Figure S1. Representative images of fibronectin micropatterns and lateral confined growth of cells on these micropatterns from Day1 to Day8. Representative control spheroid formation by overnight clumping of fibroblasts on the micropatterns. Representative alkaline phosphatase-stained images of partially reprogrammed spheroids (Day8) and control spheroids (Day1).

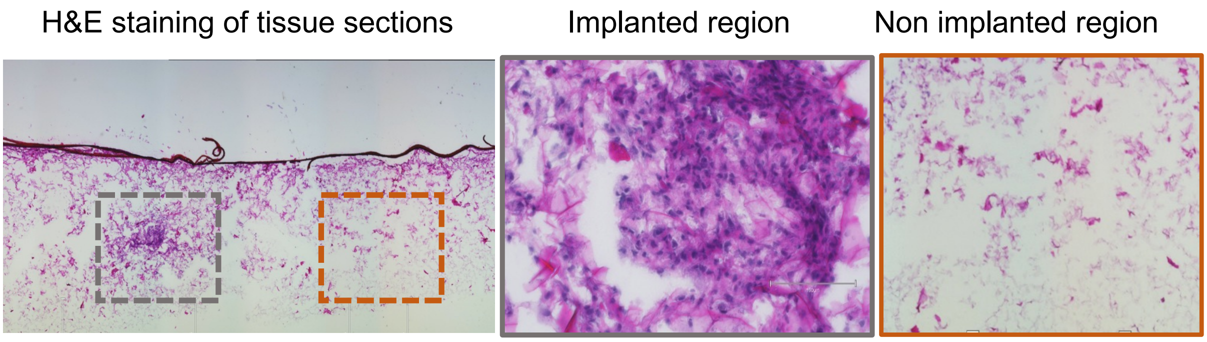

Figure S2. The representative image of H&E-stained tissue section. The grey inset represents the cell implanted region with enhanced collagen synthesis and orange inset represents the non-implanted region.

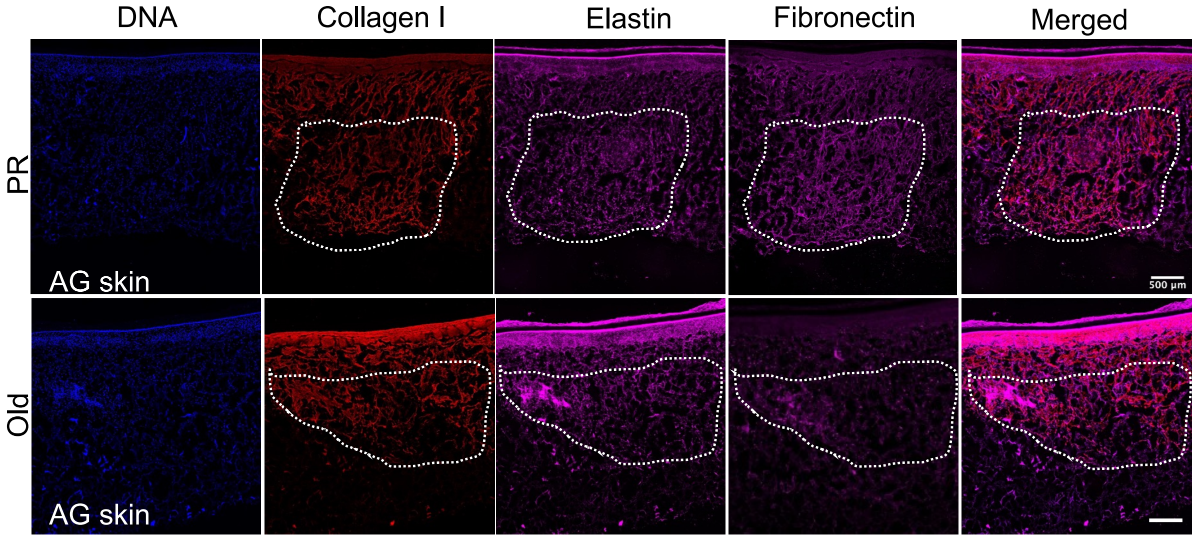

Figure S3. Representative fluorescent images (10X magnification) of histological tissue sections of in vitro aged skin tissue with different implanted cells immunostained with collagen I, elastin, and fibronectin antibodies and nucleus stained with Hoechst. scale bar is 500µm.

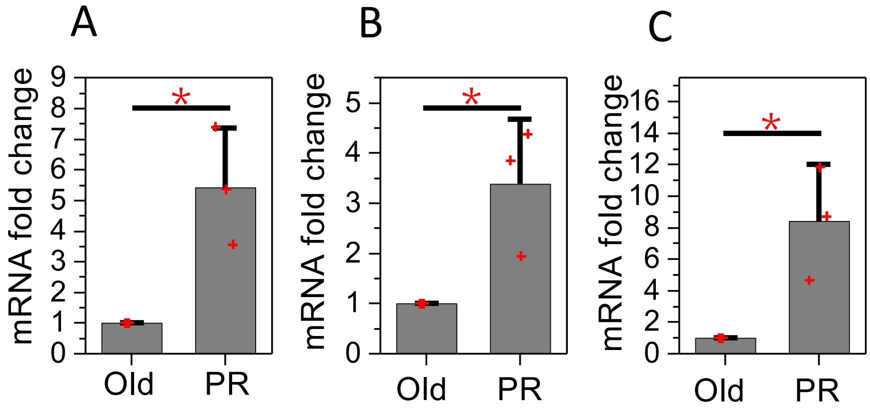

Figure S4. Fold change of the mRNA levels of Collagen I (Col1a1), Elastin (Eln) and Fibronectin (Fn1) in the implanted PR cells in aged tissue compared to old cells by qRTPCR assay. Statistical analysis for the qPCR experiments using paired student t-test. * p-value < 0.05.

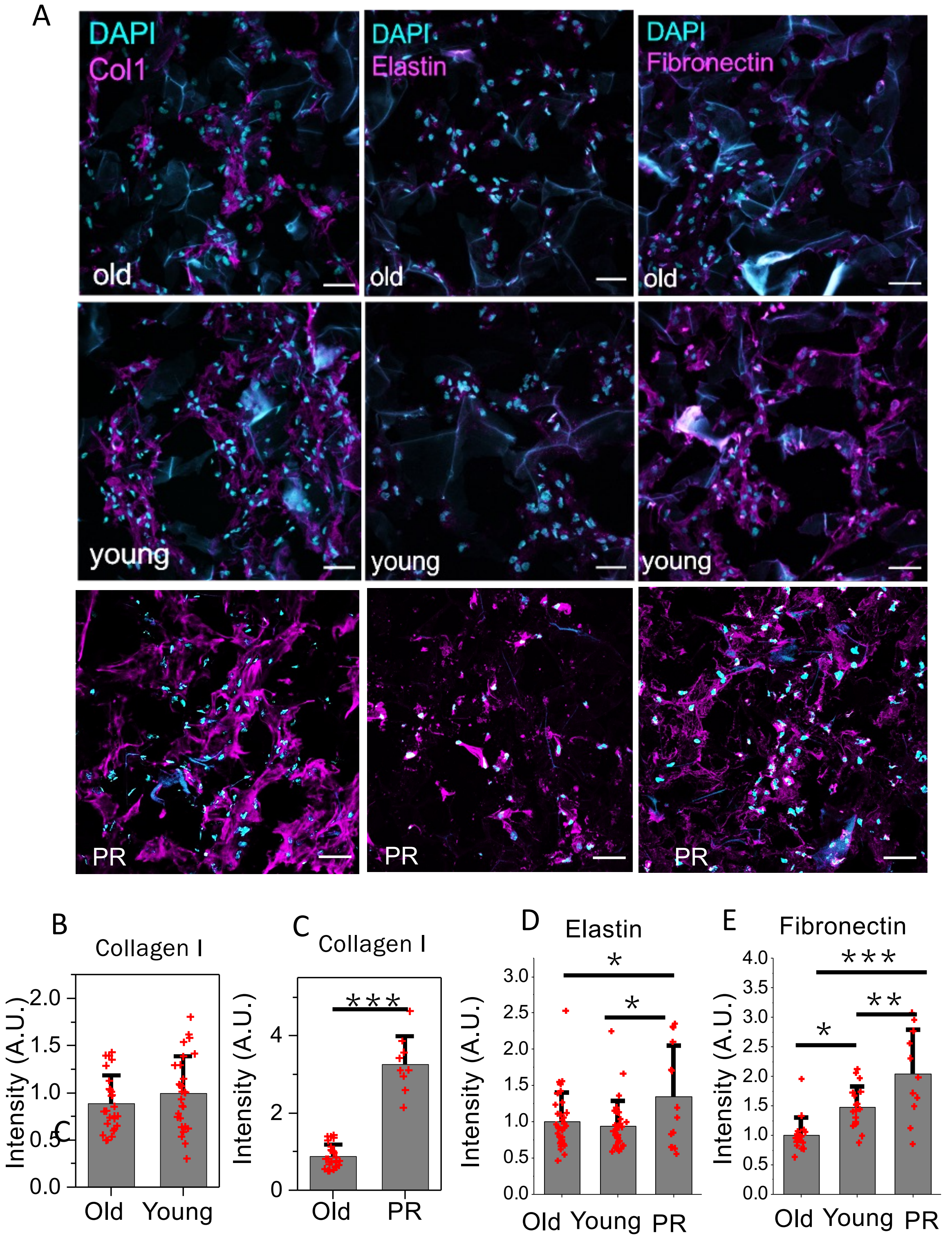

Figure S5. (A) Representative fluorescent images (40X magnification) of the immunohistological sections of the young skin tissue implanted with PR, old and young cell stained with collagen I, elastin, and fibronectin antibodies. Scale bar: 50µm. (B-E) Normalized intensity plots of the ECM proteins collagen I, elastin and fibronectin at the implanted wound regions. Statistical analysis for the quantification of fluorescent intensity (A.U.unit) among two

biological conditions (B and C) by Mann-Whitney U test and for three biological conditions (D and E) by Tukey's HSD test. * p-value < 0.05, ** p-value < 0.01, *** p-value < 0.001.

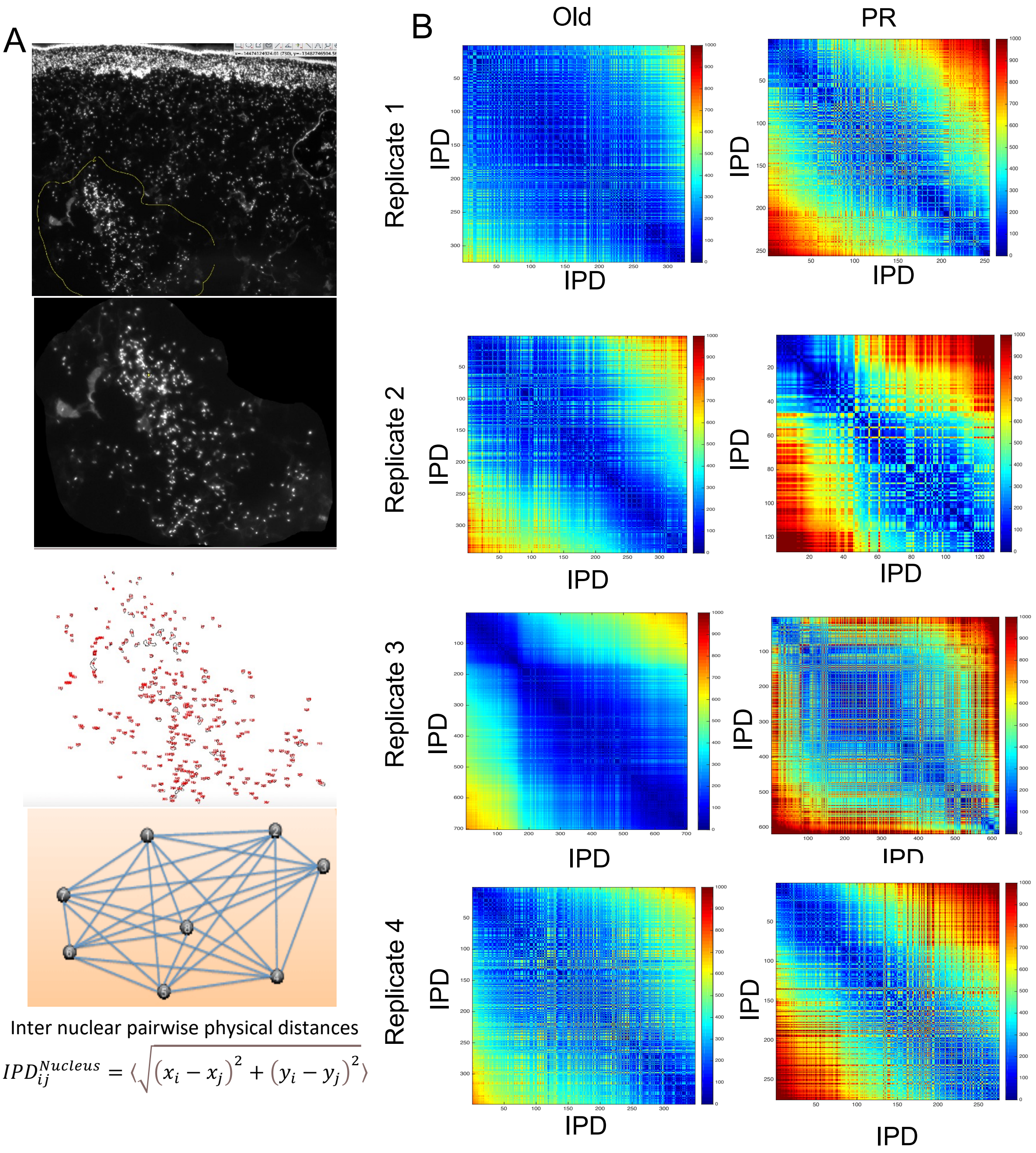

Figure S6. (A) The analysis pipeline of the inter nuclear distances of the implanted cells. (B) The different replicates of the inter physical distance (IPD) matrix of the implanted cell nucleus in histological sections. color code: warmer colors represent larger inter-chromosome distances and cooler colors smaller distances. Rows and columns indicate nuclear pairwise distances in µm.

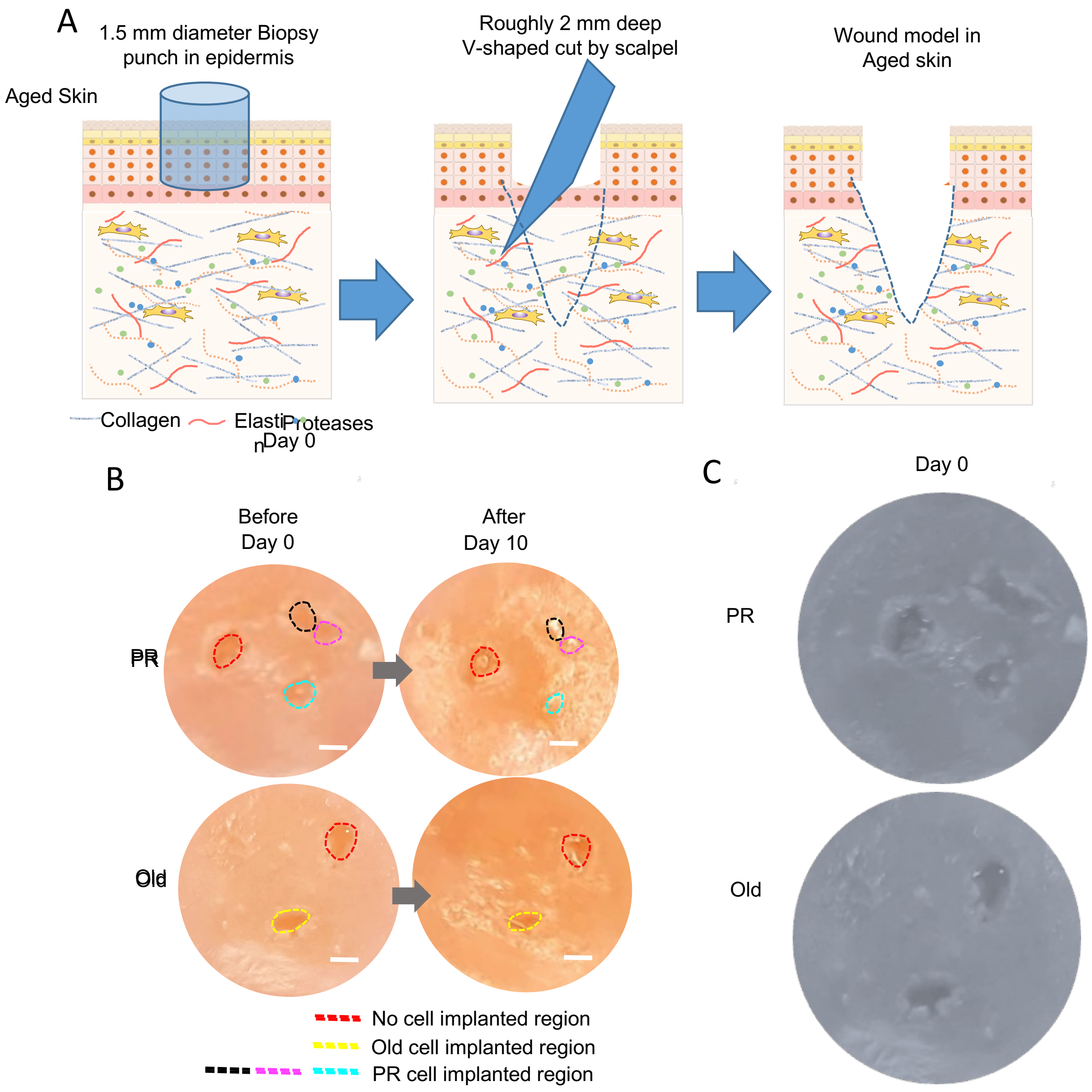

Fig. S7. (A) Schematic representation of the wounding process in aged in vitro skin. (B) Representative top surface view of the wounds on the in vitro aged skin models before and 10 days after the implanted cells. (C) Enlarged skin with wound region at day 0 b (in grey colour).

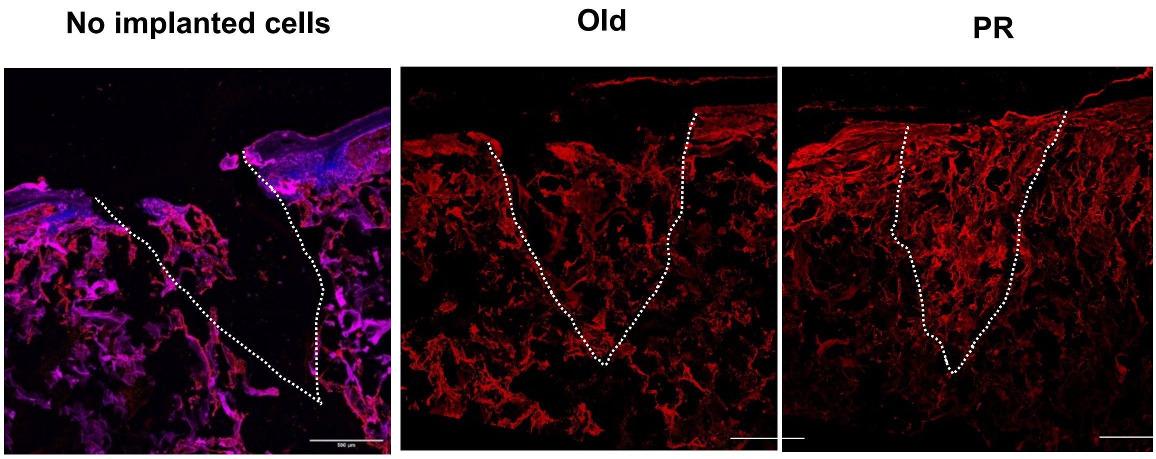

Figure S8. Representative immunofluorescence images (10X magnification) of histological tissue sections of the wound models with or without different implanted cells stained with collagen I, and nucleus stained with hoechst. scale bar is 500um. The white dotted line represents the wound sites. Same representative images of Old and PR from Figure 3C are used here to compare the non-implanted region.

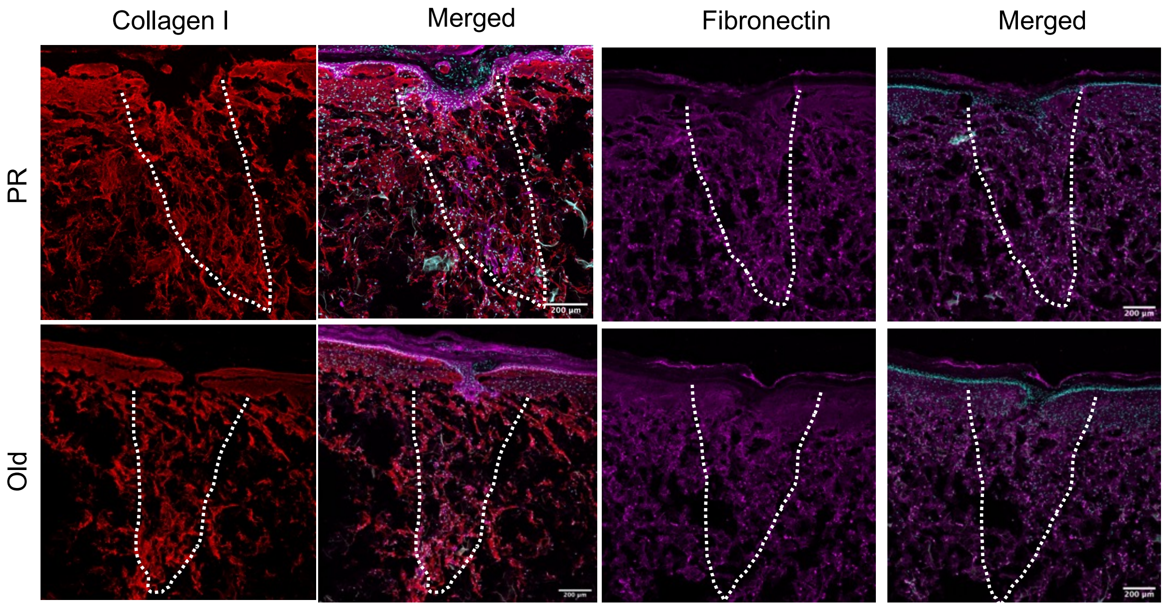

Figure S9. Representative immunofluorescence images (10X magnification) of histological tissue sections of the wound models with different implanted cells stained with collagen I and fibronectin antibody, and nucleus stained with Hoechst. scale bar is 500um. The white dotted line represents the wound sites.

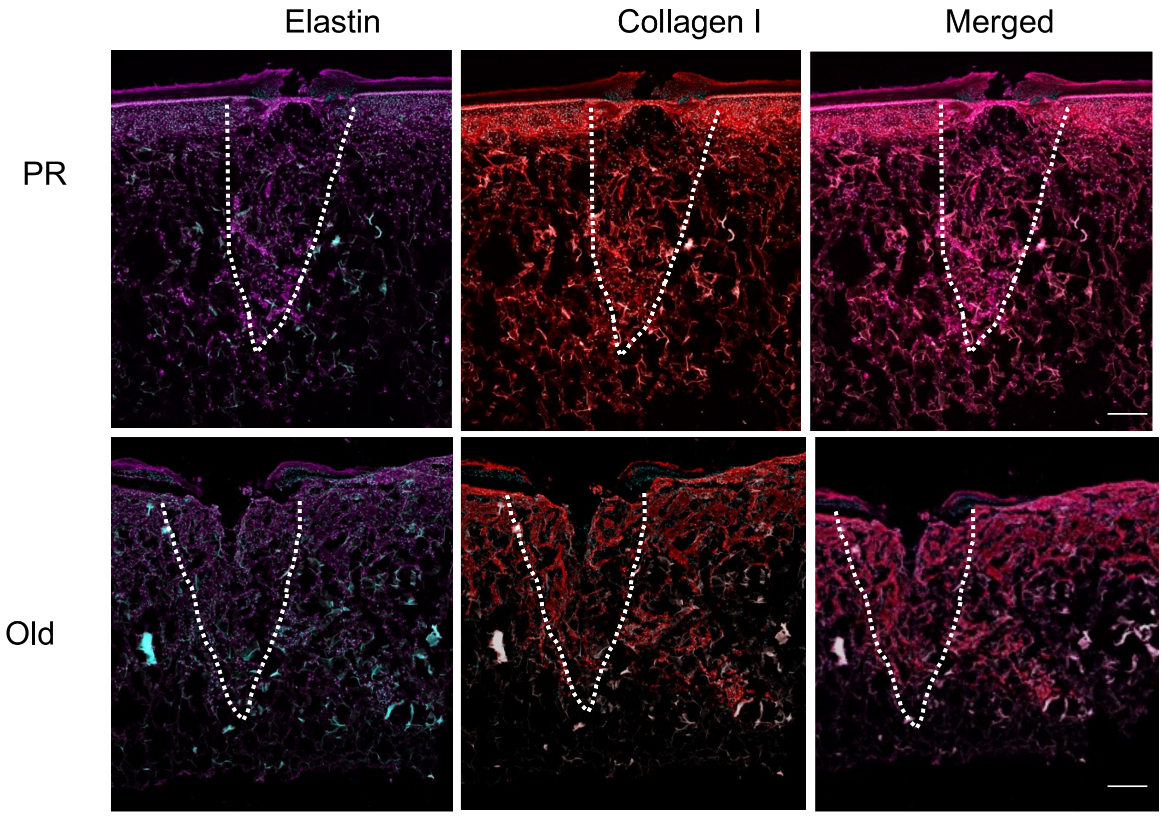

Figure S10. Representative immunofluorescence images (10X magnification) of histological tissue sections of the wound models with different implanted cells stained with elastin and aSMA antibody, and nucleus stained with Hoechst. scale bar is 500um. The white dotted line represents the wound sites.

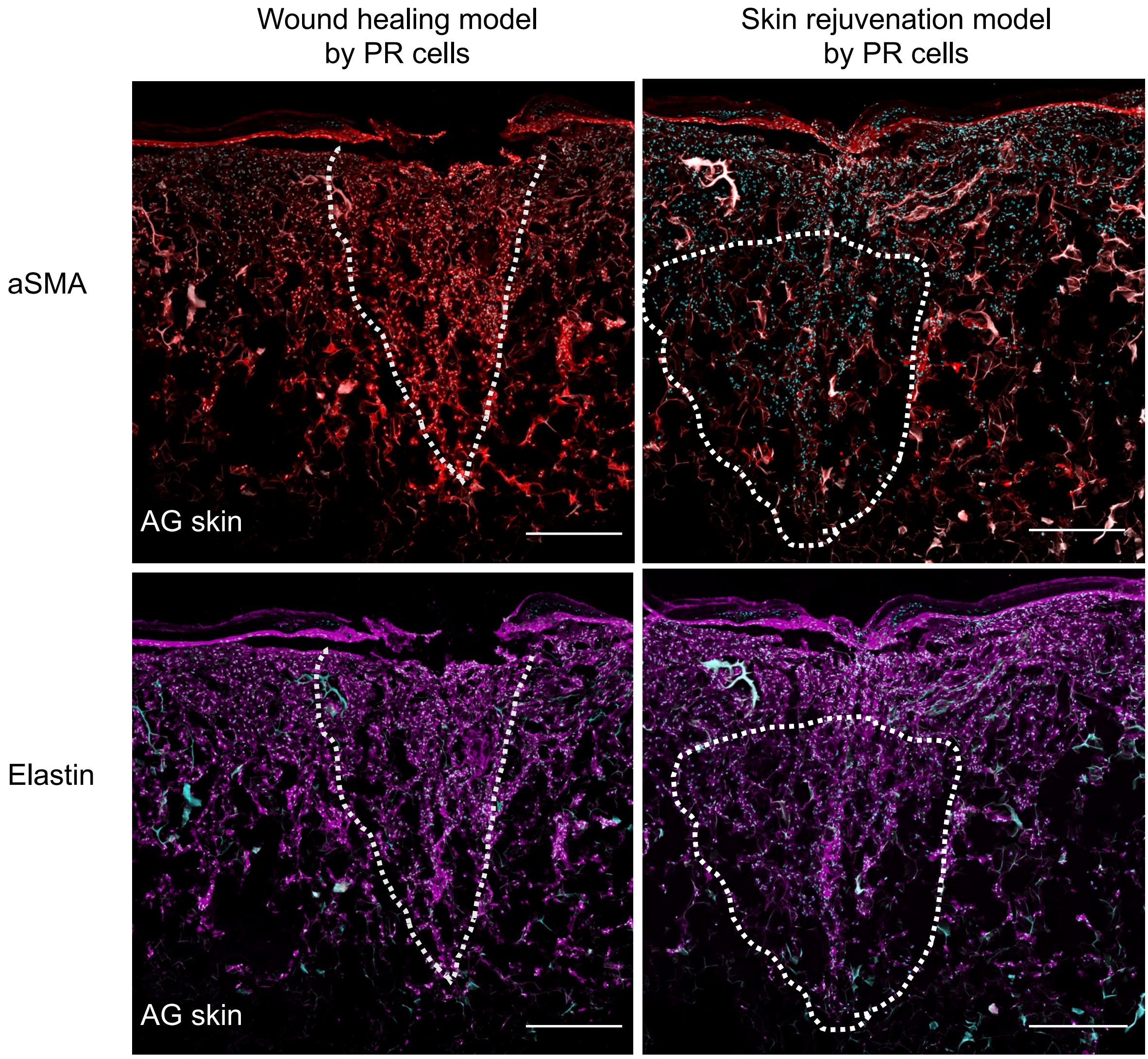

Figure S11. Representative immunofluorescence images (10X magnification) of histological tissue sections of the rejuvenation model and wound models with implanted PR cells stained with aSMA and elastin antibody, and nucleus stained with hoechst. scale bar is 500um. The white dotted line represents the cell implanted and wound sites.

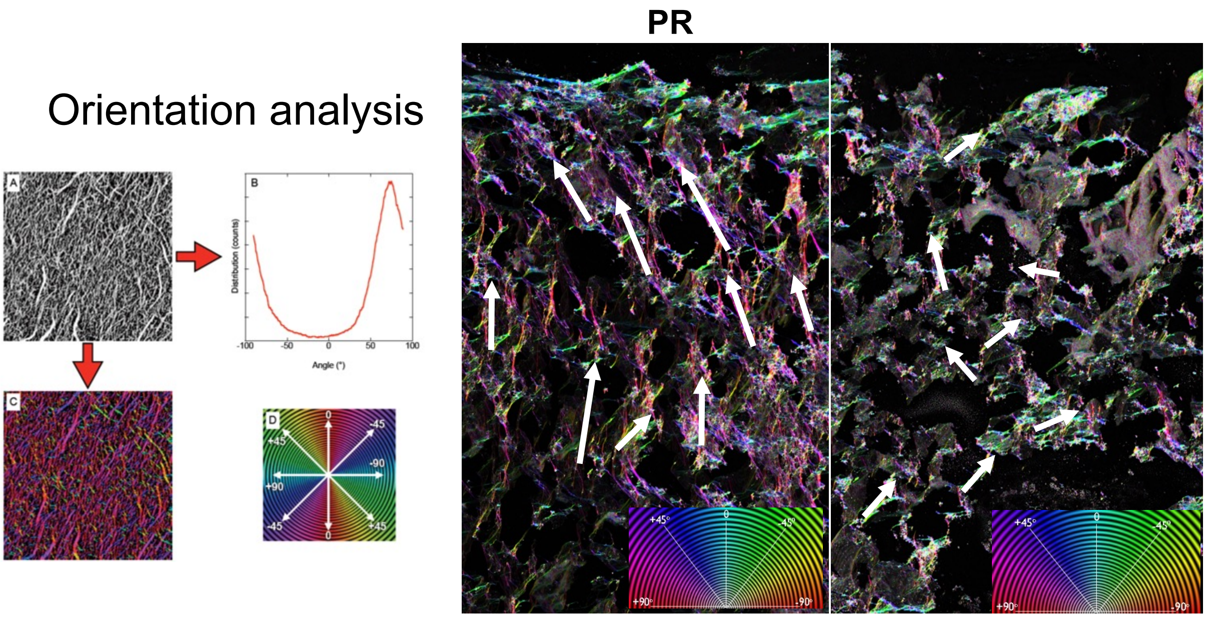

Figure S12. The fiber orientation analysis pipeline adopted from Orientation J plugin of Fiji (NIH).

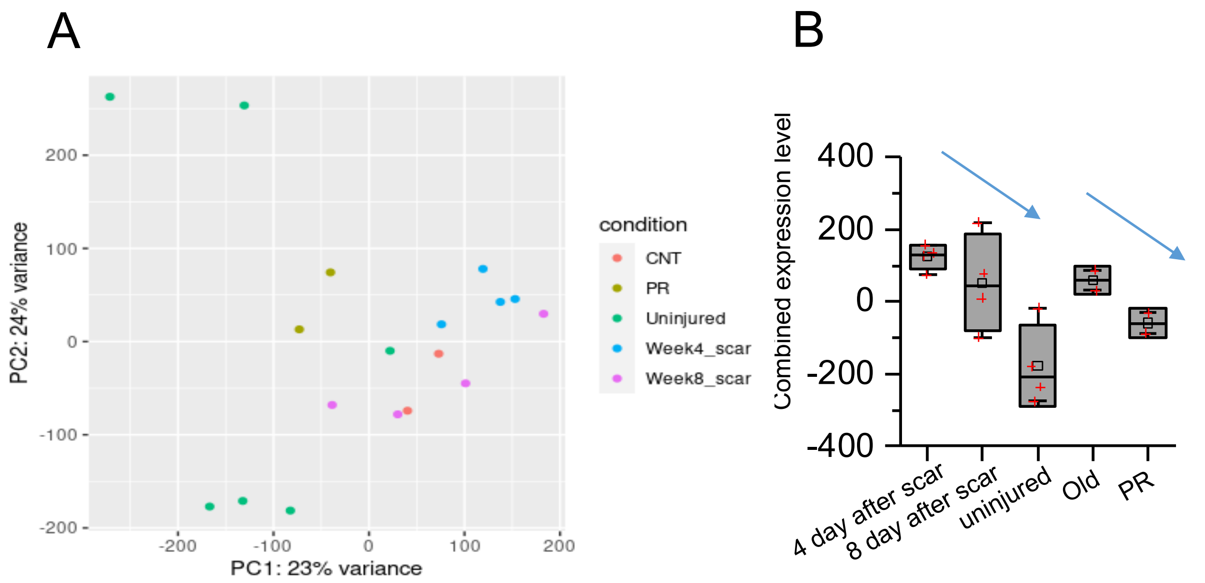

Figure S13. Comparison of wound healing response with other publicly available wound healing RNAseq data. (A) The PCA plots of the different data sets (B) Box plot of the combined expression levels (i.e. PC1).

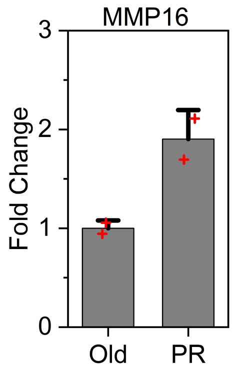

Fig. S14. Expression of Mmp16 gene in implanted PR cells compared to implanted old cells.

SUPPLEMENTARY TABLES

Table S1. Details of primary and secondary antibodies

Protein	Source	upplier and Catalog number	Applications Immunofluorescence) Overnight at 4C
Elastin	Rabbit polyclonal	Abcam (ab21610)	1:200
αSMA	Mouse monoclonal	Sigma-Aldrich (A5228)	1:100
Fibronectin	Rabbit polyclonal	Abcam (ab2413)	1:100
Collagen I	Mouse monoclonal	Abcam (ab6308)	1:200
Vimentin	Rabbit monoclonal	Cell Signalling Technologies 5741	1:200
Secondary Antibody, Alexa Fluor™ Plus 555	Goat anti-Rabbit	Invitrogen (A32732)	1:500
Secondary Antibody, Alexa Fluor™ Plus 647	Donkey anti-Mouse	Invitrogen (A32787TR)	1:500

Table S2. Primers used for qRT PCR

gene	Forward	Backward
Gapdh	AGAAGGCTGGGGCTCATTTG	AGGGGCCATCCACAGTCTTC
Fn1	GGT CAG CAT CGT TGC TCT TA	GTC CCT CGG AAC ATC AGA AA
Col1a1	GTG CGA TGA CGT GAT CTG TGA	CGG TGG TTT CTT GGT CGG T
Eln	GCA GGA GTT AAG CCC AAG G	TGT AGG GCA GTC CAT AGC CA
Acta2	GTG TTG CCC CTG AAG AGC AT	GCT GGG ACA TTG AAA GTC TCA
Vim	AGT CCA CTG AGT ACC GGA GAC	CAT TTC ACG CAT CTG GCG TTC

Mmp9	AGA CCT GGG CAG ATT CCA AAC	CGG CAA GTC TTC CGA GTA GT

Table S3. Increased secretory GF expression in the implanted PR cells.

Gene label	Gene name	log2FoldChange	adjusted p-value
IGFBP3	Insulin-like growth factor-binding protein 3	6.41	7.87E-52
NGF	Beta-nerve growth factor	3.58	7.96E-18
SCUBE3	Signal peptide, CUB and EGF-like domain-containing protein 3	7.33	1.36E-17
BDNF	Brain-derived neurotrophic factor	2.45	1.80E-11
IGFBP6	Insulin-like growth factor-binding protein 6	3.81	4.97E-09
COMP	Cartilage oligomeric matrix protein	3.16	2.42E-08
SULF1	Extracellular sulfatase Sulf-1	4.59	5.36E-07
GREM2	Gremlin-2	2.29	1.13E-06
TGFBI	Transforming growth factor-beta-induced protein ig-h3	3.82	2.21E-06
MDK	Midkine	2.14	1.17E-05
CCN2	CCN family member 2	2.67	5.62E-04
NOG	Noggin	3.28	5.62E-04
SOST	Sclerostin	6.71	5.04E-03
COL4A1	Collagen alpha-1(IV) chain	1.37	7.67E-03
GREM1	Gremlin-1	1.61	8.76E-03
IGFBP7	Insulin-like growth factor-binding protein 7	2.19	8.80E-03
VEGFA	Vascular endothelial growth factor A, long form	1.15	1.11E-02
MSTN	Growth/differentiation factor 8	2.94	2.57E-02
CRIM1	Cysteine-rich motor neuron 1 protein	1.25	3.75E-02
INHBE	Inhibin beta E chain	1.63	4.36E-02

SUPPLEMENTARY INFORMATION

Online RNAseq datasets

The NCBI-SRA accession IDs of the online RNAseq data sets used in this study are:

SRR12046127,Uninjured; SRR12046129,Week4_scar; SRR12046131,Week8_scar;
SRR12046133,Uninjured; SRR12046135,Week4_scar; SRR12046137,Week8_scar;
SRR12046139,Uninjured; SRR12046141,Week4_scar; SRR12046143,Week8_scar;
SRR12046145,Uninjured; SRR12046148,Week4_scar; SRR12046150,Week8_scar.
